# An Absolute Risk Model to Identify Individuals at Elevated Risk for Pancreatic Cancer in the General Population

**DOI:** 10.1371/journal.pone.0072311

**Published:** 2013-09-13

**Authors:** Alison P. Klein, Sara Lindström, Julie B. Mendelsohn, Emily Steplowski, Alan A. Arslan, H. Bas Bueno-de-Mesquita, Charles S. Fuchs, Steven Gallinger, Myron Gross, Kathy Helzlsouer, Elizabeth A. Holly, Eric J. Jacobs, Andrea LaCroix, Donghui Li, Margaret T. Mandelson, Sara H. Olson, Gloria M. Petersen, Harvey A. Risch, Rachael Z. Stolzenberg-Solomon, Wei Zheng, Laufey Amundadottir, Demetrius Albanes, Naomi E. Allen, William R. Bamlet, Marie-Christine Boutron-Ruault, Julie E. Buring, Paige M. Bracci, Federico Canzian, Sandra Clipp, Michelle Cotterchio, Eric J. Duell, Joanne Elena, J. Michael Gaziano, Edward L. Giovannucci, Michael Goggins, Göran Hallmans, Manal Hassan, Amy Hutchinson, David J. Hunter, Charles Kooperberg, Robert C. Kurtz, Simin Liu, Kim Overvad, Domenico Palli, Alpa V. Patel, Kari G. Rabe, Xiao-Ou Shu, Nadia Slimani, Geoffrey S. Tobias, Dimitrios Trichopoulos, Stephen K. Van Den Eeden, Paolo Vineis, Jarmo Virtamo, Jean Wactawski-Wende, Brian M. Wolpin, Herbert Yu, Kai Yu, Anne Zeleniuch-Jacquotte, Stephen J. Chanock, Robert N. Hoover, Patricia Hartge, Peter Kraft

**Affiliations:** 1 Department of Oncology, Sidney Kimmel Comprehensive Cancer Center at Johns Hopkins, Baltimore, Maryland, United States of America; 2 Department of Pathology, Sol Goldman Pancreatic Cancer Research Center, Johns Hopkins School of Medicine, Baltimore, Maryland, United States of America; 3 Department of Epidemiology, Johns Hopkins Bloomberg School of Public Health, Baltimore, Maryland, United States of America; 4 Department of Epidemiology, Harvard School of Public Health, Boston, Massachusetts, United States of America; 5 Program in Molecular and Genetic Epidemiology, Harvard School of Public Health, Boston, Massachusetts, United States of America; 6 Division of Cancer Epidemiology and Genetics, National Cancer Institute, National Institutes of Health, Department Health and Human Services, Bethesda, Maryland, United States of America; 7 Information Management Services, Silver Spring, Maryland, United States of America; 8 Department of Obstetrics and Gynecology, New York University School of Medicine, New York, New York, United States of America; 9 Department of Environmental Medicine, New York University School of Medicine, New York, New York, United States of America; 10 New York University Cancer Institute, New York, New York, United States of America; 11 National Institute for Public Health and the Environment (RIVM), Bilthoven, The Netherlands; 12 Department of Gastroenterology and Hepatology, University Medical Centre Utrecht, Utrecht, The Netherlands; 13 Department of Medical Oncology, Dana-Farber Cancer Institute, Boston, Massachusetts, United States of America; 14 Channing Laboratory, Department of Medicine, Brigham and Women's Hospital and Harvard Medical School, Boston, Massachusetts, United States of America; 15 Samuel Lunenfeld Research Institute, Mount Sinai Hospital, Toronto, Ontario, Canada; 16 Department of Laboratory Medicine/Pathology, School of Medicine, University of Minnesota, Minneapolis, Minnesota, United States of America; 17 Prevention and Research Center, Mercy Medical Center, Baltimore, Maryland, United States of America; 18 Department of Epidemiology, Johns Hopkins Bloomberg School of Public Health and Sidney Kimmel Comprehensive Cancer Center, Baltimore, Maryland, United States of America; 19 Department of Epidemiology & Biostatistics, University of California San Francisco, San Francisco, California, United States of America; 20 Department of Epidemiology, American Cancer Society, Atlanta, Georgia, United States of America; 21 Division of Public Health Sciences, Fred Hutchinson Cancer Research Center, Seattle, Washington, United States of America; 22 Department of Gastrointestinal Medical Oncology, University of Texas M.D. Anderson Cancer Center, Houston, Texas, United States of America; 23 Group Health Center for Health Studies, Seattle, Washington, United States of America; 24 Department of Epidemiology and Biostatistics, Memorial Sloan-Kettering Cancer Center, New York, New York, United States of America; 25 Department of Health Sciences Research, Mayo Clinic, Rochester, Minnesota, United States of America; 26 Department of Epidemiology and Public Health, Yale University School of Public Health, School of Medicine, New Haven, Connecticut, United States of America; 27 Department of Medicine and Vanderbilt-Ingram Cancer Center, Vanderbilt University, Nashville, Tennessee, United States of America; 28 Cancer Epidemiology Unit, University of Oxford, Nuffield department of Clinical Medicine, Oxford, United Kingdom; 29 UK Biobank, Oxford, United Kingdom; 30 Inserm, (Institut National de la Santé et de la Recherche Médicale) and Institut Gustave Roussy, Villejuif, France; 31 Divisions of Preventive Medicine and Aging, Department of Medicine, Brigham and Women's Hospital and Harvard Medical School, Boston, Massachusetts, United States of America; 32 Department of Ambulatory Care and Prevention, Harvard Medical School, Boston, Massachusetts, United States of America; 33 Division of Cancer Epidemiology, German Cancer Research Center (DKFZ), Heidelberg, Germany; 34 George W. Comstock Center for Public Health Research and Prevention, Hagerstown, Maryland, United States of America; 35 Cancer Care Ontario and Dalla Lana School of Public Health, University of Toronto, Toronto, Ontario, Canada; 36 Unit of Nutrition, Environment and Cancer, Catalan Institute of Oncology (ICO-IDIBELL), Barcelona, Spain; 37 Division of Cancer Control and Population Science, National Cancer Institute, National Institutes of Health, Department of Health and Human Services, Bethesda, Maryland, United States of America; 38 Massachusetts Veterans Epidemiology Research and Information Center, Veterans Affairs Boston Healthcare System, Boston, Massachusetts, United States of America; 39 Department of Nutrition, Harvard School of Public Health, Boston, Massachusetts, United States of America; 40 Departments of Oncology, Pathology and Medicine, The Johns Hopkins University School of Medicine, Baltimore, Maryland, United States of America; 41 Department of Public Health and Clinical Medicine, Nutritional Research, Umeå University, Umeå, Sweden; 42 Core Genotyping Facility, SAIC-Frederick Inc., NCI-Frederick, Frederick, Maryland, United States of America; 43 Program in Biostatistics and Biomathematics, Division of Public Health, Fred Hutchinson Cancer Research Center, Seattle, Washington, Massachusetts, United States of America; 44 Department of Medicine, Memorial Sloan-Kettering Cancer Center, New York, New York, United States of America; 45 Departments of Epidemiology, Medicine and Obstetrics & Gynecology Director, Center for Metabolic Disease Prevention, University of California Los Angeles, Los Angeles, California, United States of America; 46 Section of Epidemiology, Department of Public Health, Aarhus University, Aarhus, Denmark; 47 Molecular and Nutritional Epidemiology Unit, Cancer Research and Prevention Institute - ISPO Florence, Italy; 48 International Agency for Research on Cancer, Lyon, France; 49 Bureau of Epidemiologic Research, Academy of Athens, Athens, Greece; 50 Division of Research, Kaiser Permanente, Northern California Region, Oakland, California, United States of America; 51 Imperial College London, London, United Kingdom; 52 Department of Chronic Disease Prevention, National Institute for Health and Welfare, Helsinki, Finland; 53 Department of Social and Preventive Medicine, University at Buffalo, State University of New York Buffalo, Buffalo, New York, United States of America; 54 Department of Biostatistics, Harvard School of Public Health, Boston, Massachusetts, United States of America; Centro Nacional de Investigaciones Oncológicas (CNIO), Spain

## Abstract

**Purpose:**

We developed an absolute risk model to identify individuals in the general population at elevated risk of pancreatic cancer.

**Patients and Methods:**

Using data on 3,349 cases and 3,654 controls from the PanScan Consortium, we developed a relative risk model for men and women of European ancestry based on non-genetic and genetic risk factors for pancreatic cancer. We estimated absolute risks based on these relative risks and population incidence rates.

**Results:**

Our risk model included current smoking (multivariable adjusted odds ratio (OR) and 95% confidence interval: 2.20 [1.84–2.62]), heavy alcohol use (>3 drinks/day) (OR: 1.45 [1.19–1.76]), obesity (body mass index >30 kg/m^2^) (OR: 1.26 [1.09–1.45]), diabetes >3 years (nested case-control OR: 1.57 [1.13–2.18], case-control OR: 1.80 [1.40–2.32]), family history of pancreatic cancer (OR: 1.60 [1.20–2.12]), non-O ABO genotype (AO vs. OO genotype) (OR: 1.23 [1.10–1.37]) to (BB vs. OO genotype) (OR 1.58 [0.97–2.59]), rs3790844(chr1q32.1) (OR: 1.29 [1.19–1.40]), rs401681(5p15.33) (OR: 1.18 [1.10–1.26]) and rs9543325(13q22.1) (OR: 1.27 [1.18–1.36]). The areas under the ROC curve for risk models including only non-genetic factors, only genetic factors, and both non-genetic and genetic factors were 58%, 57% and 61%, respectively. We estimate that fewer than 3/1,000 U.S. non-Hispanic whites have more than a 5% predicted lifetime absolute risk.

**Conclusion:**

Although absolute risk modeling using established risk factors may help to identify a group of individuals at higher than average risk of pancreatic cancer, the immediate clinical utility of our model is limited. However, a risk model can increase awareness of the various risk factors for pancreatic cancer, including modifiable behaviors.

## Introduction

Pancreatic Cancer is the 4^th^ leading cause of cancer death in the United States [1]. While the lifetime risk (age 85) of pancreatic cancer for US Caucasians is only 1.5% [1], the five-year survival rate is less than 4.8%, the poorest of any major tumor type [1]. The primary reason for the poor survival rate is the high proportion of patients (>80%) who are diagnosed with locally advanced or metastatic disease. However, five-year survival rates for patients with early-stage resectable disease can exceed 20% [1], [2], underscoring the need to improve early detection. Numerous studies are underway to identify and validate promising biomarkers [3], [4] for early detection. In addition, several clinical studies have shown that imaging via e ndoscopic ultrasound, MRI or CT scan can detect pre-cancerous changes in the pancreas among high-risk individuals [5]–[8].

Given the low incidence of pancreatic cancer in the general population, widespread screening may not be practically feasible, even with a highly sensitive and specific test. Therefore, identification of individuals with substantially elevated risk will be important to the success of early detection studies. Pancreatic cancer tends to cluster in families and the heritability has been estimated to 0.36, indicating a strong genetic influence [9]. Although high-penetrance germline mutations have been identified, they only explain a small fraction of cases (less than 5%), indicating that many susceptibility variants (rare and common) remains to be identified. There appears to be no demographic differences between sporadic and familial pancreatic cancers. While there has been some suggestion that familial pancreatic cancers may have a slightly earlier age-of-onset (approximately 5 years) this finding has been inconsistent [10], [11]. No differences in the pathology of invasive pancreatic cancers in patients with familial vs non-familial pancreatic cancers have been reported [12] (A. Klein, unpublished work). However, non-invasive precursors are more common in patients with familial pancreatic cancer and these precursor lesions of higher-grade than the lesions that occur in patients without a family history [12]

Pancreatic cancer risk has been associated with cigarette smoking [13], heavy alcohol use [14], [15], diabetes mellitus [16], increased body mass index [17], family history of pancreatic cancer [18] and inherited genetic variation. Germline mutations in several genes, *BRCA2*, *PALB2*, *p16*, *ATM*, *STK11*, *PRSS1*, *SPINK1* and DNA mis-match repair, have been associated with an increased pancreatic cancer risk [19]–[26]. In addition, two recently completed genome-wide association studies (GWAS), PanScan1 and PanScan2, have identified variants in *ABO* (rs505922), 1q32.1 (rs3790844), 13q22.1 (rs9543325) and 5p15.3 (rs401681) that are associated with a modestly increased risks of pancreatic cancer [27], [28]. The *ABO* single nucleotide polymorphism (SNP) rs505922 is in strong linkage disequilibrium with O/non-O blood group alleles indicating that individuals with non-O blood groups are at an increased risk of developing pancreatic cancer [29], [30]. In addition, haplotypes of SNPs rs505922 and rs8176746 are perfectly correlated with the O and B alleles, respectively [29], [30], and the assessment of both SNPs allow for complete discrimination between blood groups.

The aim of this study was to derive an absolute risk model for pancreatic cancer in the general population. By using data from both prospective cohort studies and retrospective case-control studies, we developed a relative risk model that included established risk factors for pancreatic cancer. We then estimated participants' absolute risk of developing pancreatic cancer by combining the derived risk model with incidence data from the SEER registries.

## Methods

### Study Population

The PanScan Consortium is comprised of 12 case-control studies nested within prospective cohorts and 8 retrospective case-control studies that participated in two GWAS of pancreatic cancer [27], [28]. The cohorts include: The Alpha-Tocopherol Beta-Carotene Prevention Study (ATBC), Give us a Clue to Cancer and Heart Disease Study (CLUEII), Cancer Prevention Study (CPSII), European Prospective Investigation Into Cancer and Nutrition Study (EPIC), Health Professionals Follow-Up Study (HPFS), Nurses' Health Study (NHS), The New York University, Women's Health Study (NYU-WHS), Physicians Health Study (PHS), Prostate, Lung, Colorectal Ovarian Cancer Screening Trial (PLCO), Shanghai Men's and Women's Health Study (SMWHS), Women's Health Initiative (WHI), and the Women's Health Study (WHS). The retrospective case-control studies were conducted at the Mayo Clinic, Yale University (Connecticut Pancreas Cancer Case Control Study), Group Health (Seattle Puget Sound) and Kaiser Permanente in Northern California (PACIFIC Study), Memorial Sloan Kettering Cancer Center, MD Anderson Cancer Center, University of California San Francisco, Johns Hopkins Medical School, and Mount Sinai Toronto.

### Ethics Statement

The Institutional Review Boards approval, including approval of the consent procedure, was obtained for each of the studies as follows: ATBC and Ag.Health (National Cancer Institute Special Studies Institutional Review Board (SSIRB)), CLUE (Johns Hopkins School of Public Health (JHSPH) Institutional Review Board Office), CPS II (Emory University Institutional Review Board), EPIC (International Agency for Research on Cancer (IARC) Institutional Review Board Office), HPFS, WHS, NHS and PHS (Partners Healthcare System, Human Research Committee, Partners Human Research Office), PLCO (National Cancer Institute Special Studies Institutional Review Board (SSIRB)), SMWHS (Vanderbilt University Institutional Review Board), WHI (Fred Hutchinson Cancer Research Center Institutional Review Board), Group Health – PACIFIC (Group Health Research Institute, Human Subjects Review Office), JHU (Johns Hopkins Medicine, Office of Human Subjects Research, Institutional Review Board), MAYO (Mayo Clinic Institutional Review Board), MDA (MD Anderson Cancer Center, Office of Protocol Research, Institutional Review Board), MSKCC (Memorial Sloan-Kettering Cancer Center, Institutional Review Board/Privacy Board), TORONTO (University Health Network, Research Ethics Board),(UCSF) University of California San Francisco, Human Research Protection Program, Committee on Human Research, YALE (Yale University, Human Investigation Committee). Written consent was obtained from all study participants. In addition, because the National Cancer Institute is the coordinating center for the PanScan I and II studies, the National Cancer Institute Special Studies Institutional Review Board (SSIRB) reviewed and approved the PanScan protocol in its entirety.

A brief description of each study is provided in Tables S1 and S2. Genotype and covariate data were available for 3,851 cases and 3,924 controls. Analyses were restricted to non-Hispanic whites as four percent of study participants reported non-European ancestry (n = 493), precluding meaningful analyses within this subgroup. Participants with diabetes diagnosed (n = 467) within 3 years of pancreatic cancer diagnosis were excluded because of possible reverse causation. To ascertain potential confounding effects of diabetes proximal to pancreatic cancer diagnosis, we conducted sensitivity analyses including/excluding these participants as well as modeling an indicator variable denoting diabetes diagnosis within three years prior to pancreatic cancer diagnosis. Point estimates for the other key risk factors were not substantially changed among the models. A total of 3,349 cases and 3,654 controls were included in our analyses.

### Description of covariate and SNP data

For each study, we collected information on age, sex, ethnicity, cigarette smoking history (never/former/current), history of diabetes mellitus (never/>3 years duration), body mass index (BMI, ≤30/>30), heavy alcohol consumption (≤3 drinks per day/>3 drinks per day), and family history of pancreatic cancer (yes/no). Age was defined as age at diagnosis for cases and age at interview for controls (Table 1). The following criteria were used to select risk factors for inclusion in the model 1) factor has been consistently associated with pancreatic cancer risk and 2) data was available from both the case-control and cohort studies. Missing covariate data were modeled using the missing indicator method where a separate ‘missing’ level is created within each covariate. Details on data collection for the various covariates have been described in previous publications [15], [17], [31], [32] Genotyping in PanScan has been described earlier [27], [28]. ABO alleles were derived from genotypes for rs505922 and rs8176746 as described previously [30]. Complete case analysis was conducted for the genotype data; the small number of participants for whom data were missing on at least one of the genetic markers (n = 6) were excluded from any analyses that included genetic risk factors.

**Table 1:** Participants' Characteristics, the PanScan Consortium.

Characteristic	Cohort		Case-Control	
	Cases n (%)	Controls n (%)	Cases n (%)	Controls n (%)
**Sex**				
Male	676 (50)	732 (51)	1076 (54)	1171 (53)
Female	667 (50)	713 (49)	930 (46)	1038 (47)
**Age in years categories**				
<50	39 (3)	20 (1)	184 (9)	219 (10)
51–60	185 (14)	161 (11)	498 (25)	494 (22)
61–65	241 (18)	239 (17)	339 (17)	361 (16)
66–70	290 (22)	349 (24)	328 (16)	366 (16)
71–75	290 (22)	349(24)	299 (15)	369 (17)
76–80	200(15)	224(16)	225 (11)	270 (12)
81+	98(7)	103(7)	133 (7)	130 (6)
**Cigarette smoking status**				
Never smoker	768(59)	902(63)	661 (39)	900 (48)
Former smoker	357(27)	405(28)	772 (45)	820 (44)
Current smoker	176(14)	116(8)	270 (15)	154 (8)
Missing/Not Available	42	22	293	315
**Diabetes mellitus**				
Never	1147(93)	1309 (95)	1103 (86)	1352 (91)
>3 years duration	89 (7)	67 (5)	181 (14)	130 (9)
Unknown	107	69	722	727
**Family history of pancreatic cancer**				
No	524 (94)	577 (97)	1507 (94)	1620 (96)
Yes	33 (6)	20 (3)	89 (6)	66 (4)
Missing/Not Available	786	848	410	523
**Heavy Alcohol Use (>3 drinks per day)**				
No	1083 (92)	1188 (94)	942 (85)	1224 (90)
Yes	99 (8)	82 (6)	168 (15)	136 (10)
Missing/Not Available	161	175	896	849
**Body Mass Index**				
<18.5	10 (1)	17 (1)	16(1)	17(1)
18.5–25	499 (38)	585 (41)	574(38)	695(40)
25–30	565 (42)	566 (39)	606(40)	723(42)
>30	256 (19)	267 (19)	323(21)	287(17)
Missing/Not Available	13	10	487	487
**ABO genotype**				
O/O	449 (33)	603 (42)	772 (38)	961 (44)
A/O	493 (37)	498 (34)	728 (36)	773 (35)
A/A	135 (10)	102 (7)	167 (8)	159 (7)
B/O	163 (12)	152 (11)	221 (11)	215 (10)
B/B	20 (2)	11 (1)	17 (1)	19 (1)
A/B	81 (6)	78 (5)	97 (5)	80 (4)
**1q32 rs3790844**				
T/T	835 (62)	817 (57)	1319 (66)	1273 (58)
T/C	436 (32)	534 (37)	605 (30)	798 (36)
C/C	72 (5)	94 (7)	82 (4)	137 (6)
**5p15 rs401681**				
C/C	376 (28)	434 (30)	506 (25)	698 (32)
C/T	649 (48)	688 (48)	1029 (51)	1098 (50)
T/T	318 (24)	323 (22)	471 (23)	413 (19)
**13q22 rs9543325**				
T/T	448(33)	573 (40)	670 (33)	879 (40)
T/C	672(50)	683 (47)	952 (47)	1027 (47)
C/C	223(17)	189 (13)	382 (19)	302 (14)

### Statistical Methods

Before pooling data from the cohort and case-control studies, logistic regression models were fit separately to both the case-control and cohort data. We compared OR estimates for each risk factor from the case-control and cohort studies and looked for substantive differences. With the exception of history of diabetes mellitus, no substantive differences were observed. Data were pooled in the subsequent analysis.

To build a relative risk model for pancreatic cancer, we fit a logistic regression model for case-control status as a function of smoking history, history of diabetes, family history of pancreatic cancer, alcohol consumption, obesity and GWAS-identified risk markers including ABO blood group, adjusted for sex, age and study. In particular, we fit the following logistic regression model:
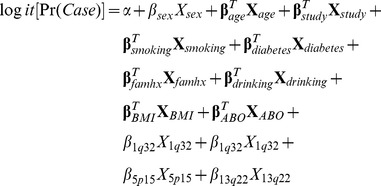



The terms **X** *_age_*, **X** *_study_*, **X** *_smoking_ et cetera* are vectors of categorical indicator variables, corresponding to the categories in Tables 1 and 2. For example, a former smoker would have **X** *_smoker_* = (1,0,0)^T^, while a never smoker would have **X** *_smoker_* = (0,0,0)^T^. The SNPs *X_1q32_*, *X_5p15 and_ X_13q22_* were coded as counts of risk alleles, and *X_sex_* was an indicator for female sex. We modeled the effect of history of diabetes mellitus separately for retrospective case-control and prospective nested case-control studies.

**Table 2:** Association between pancreatic cancer risk and smoking, personal history of diabetes, family history of pancreatic cancer, alcohol use, body mass index, and known genetic markers.

Characteristic	Multivariate Odds Ratio (95% CI) Final Model
**Cigarette smoking**	
Never	1.00
Former	1.22 (1.09, 1.37)
Current	2.20 (1.84, 2.62)
**Diabetes mellitus**	
Never	1.00
>3 years duration (cohort studies)	1.62 (1.15, 2.28)
Unknown (cohort studies)	2.37 (1.64, 3.44)
>3 years duration (case-control studies)	1.77 (1.37, 2.31)
Unknown (case-control studies)	1.10 (0.80, 1.50)
**Family history of pancreatic cancer**	
No	
Yes	1.00
**Heavy alcohol use (>3 drinks per day)**	1.60 (1.20, 2.12)
No	1.00
Yes	1.45 (1.19, 1.76)
**Body mass index**	
<18.5	0.91 (0.54, 1.53)
18.5–25	1.00
25–30	1.08 (0.96, 1.22)
>30	1.26 (1.09, 1.45)
**ABO genotype**	
OO	1.00
AO	1.23 (1.10, 1.37)
AA	1.49 (1.24, 1.79)
BO	1.35 (1.15, 1.59)
BB	1.58 (0.97, 2.59)
AB	1.44 (1.15, 1.81)
**1q32 rs3790844 (per risk allele)**	1.29 (1.19, 1.40)
**5p15 rs401681 (per risk allele)**	1.18 (1.10, 1.26)
**13q22 rs9543325 (per risk allele)**	1.27 (1.18, 1.36)

Given estimates of the log odds ratios, we calculated the relative risk for an individual with a specific risk profile 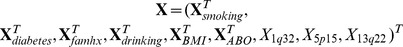 as follows: 
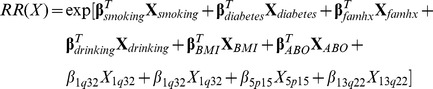



This relative risk model was then used to calculate Receiver Operating Characteristic (ROC) curves (by comparing the distribution of ORs in cases versus controls) and age-specific incidence rates (described below). We also fit relative risk models a) excluding the genetic factors and b) including only the genetic factors, in order to compare the relative contribution of genetic and non-genetic factors to risk prediction.

We calculated the area under the ROC curves using the Mann-Whitney statistic and compared the areas for different models using the method described by DeLong et al. [33] as implemented in SAS PROC LOGISTIC. These calculations were performed in the subset of data with no missing genetic or non-genetic covariate data (435 cases and 458 controls from the cohort studies and 885 cases and 1,093 controls from the case-control studies).

Age-specific incidence for an individual with risk factor profile **X** was calculated as *ρ_SEX_*(*t*) RR(**X**), where the sex-specific baseline incidence rate *ρ_SEX_*(*t*) was calculated as the appropriate sex-and age-specific average incidence rate divided by the average relative risk in controls with no missing covariate data [34]. Average incidence rates by age for white men and women were based on SEER (Surveillance, Epidemiology and End Results, http://seer.cancer.gov/) data for years 2000–2008 (SEER17). The baseline incidence was the incidence among participants who had never smoked, had never been diagnosed with diabetes, had no family history of pancreatic cancer, drank an average of ≤3 alcoholic drinks/day, had an adult BMI between 18.5 and 25, and did not carry any of the risk alleles at the four known risk loci. Lifetime risks were calculated by integrating the age-specific incidence rates, accounting for mortality due to other causes [34], [35].

To examine the value of adding genotype data to a classic non-genetic risk prediction tool, we plotted the estimated lifetime risk for cases and controls based on a model without genetic factors and a model with genetic factors. We also calculated the net reclassification index (NRI) for men and women separately, using twice the average lifetime risk to define high and low risk categories [36], [37].

## Results

Demographic and risk factor characteristics of study participants are presented in Table 1. Multivariable adjusted odds ratios (OR) are presented in Table 2 for the association between the risk factors included in our model and pancreatic cancer. In our study population, current smoking was associated with an increased risk of pancreatic cancer (OR: 2.21, 95% confidence interval [CI] 1.85, 2.64) as were heavy alcohol use (OR 1.37, 95%CI 1.12, 1.68), BMI >30 (OR 1.20, 95%CI 1.04, 1.40), diabetes of >3 year duration (cohort OR 1.62, 95%CI 1.15, 2.28; case-control OR: 1.77, 95%CI: 1.37, 2.31) and family history of pancreatic cancer (OR 1.58, 95%CI: 1.19, 2.11). In addition, all four genetic variants tested were associated with pancreatic cancer (OR for non-O ABO genotypes ranged from 1.25 to 1.58, and the per-allele odds ratios for the other three risk SNPs ranged from 1.18 to 1.49).

The area under the ROC curve (AUROC) for a risk model including only genetic factors was 57% (95%CI 0.55–0.59), whereas the AUROC for a model including only non-genetic factors was 58% (95%CI 0.56–0.60). The AUROC for a model including both genetic and non-genetic factors was 61% (95%CI 0.58–0.63), which was statistically significantly larger than both the model including only non-genetic factors and the model including only genetic factors (p<0.0001).

**Figure 1:**
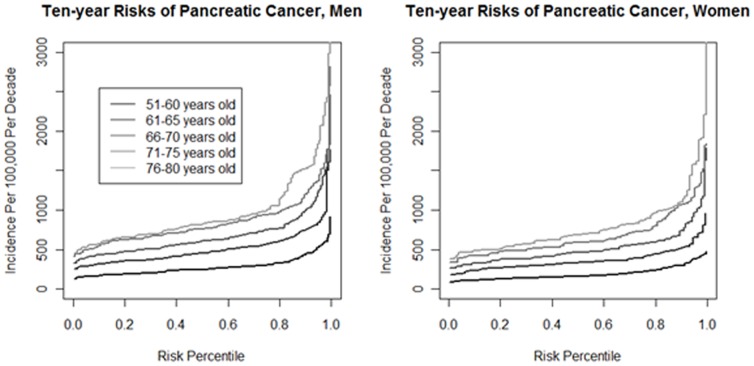
Ten-year risks of pancreatic cancer (y-axis), by age, gender, and risk score percentile (x-axis). The risk score includes smoking history, heavy alcohol intake, BMI, history of diabetes, family history of pancreatic cancer, ABO genotype and three common genetic variants associated with pancreatic cancer.

Figure 1 displays the ten-year risks of pancreatic cancer for men and women in different age categories (51–60, 61–65, 66–70, 71–75, and 76–80) as a function of risk percentile based on a model including all risk factors (see Methods). This figure demonstrates the importance of age as predictor of pancreatic cancer risk, with risk increasing with increasing age. Only a few individuals had a 10 year absolute risk greater than 2% even if all genetic and non-genetic risk factors were present.

**Figure 2:**
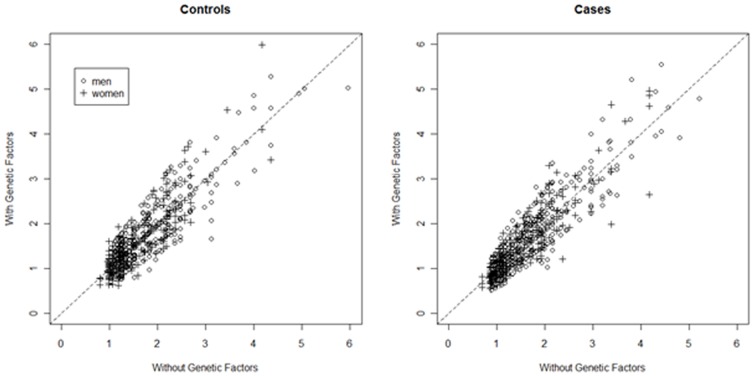
Reclassification of lifetime risk of pancreatic cancer among cohort controls after adding genetic information to the risk model.

Figure 2 shows the distribution of estimated lifetime risks for models that include or do not include genetic factors. Individual risks varied slightly depending on which model was used to estimate them. The median difference in lifetime risk estimates from the model with genetics to the model without genetics was 0.0% (inter-quartile range −0.2% to 0.2%) for both male and female controls. The NRI comparing the risk model with genetics to the risk model with no genetic factors was −0.010±0.0.008 and −0.020±0.011 for men and women respectively (Table 3). Neither of these estimates was statistically significant (one-sided *p* = 0.89 and *p* = 0.97, respectively), suggesting that adding genetic factors to the risk model did not improve clinical utility (defined as the ability to correctly classify individuals at twice average risk).

**Table 3:** Reclassification of lifetime risk of pancreatic cancer after adding genetic information to the risk model with both genetic and non-genetic covariates.

		Men		Women	
	Risk model with non-genetic covariates only	Less than twice average lifetime risk (≤2.94%)	More than twice average lifetime risk (>2.94%)	Less than twice average lifetime risk (≤2.62%)	More than twice average lifetime risk (>2.62%)
Controls	Less than twice	858	11	620	20
	average risk				
	More than twice	9	20	2	7
	average risk				
Cases	Less than twice	716	10	517	11
	average risk				
	More than twice	16	28	7	15
	average risk				

As expected, considering that we included established risk factors for pancreatic cancer in our non-genetic risk model, this model improved classification relative to a null model that classified individuals according to their sex-specific average. The NRI comparing the model with non-genetic risk factors alone to this null model was 0.025±0.010 (one-sided *p* = *0.009*) for men and 0.026±0.010 (one-sided *p = 0.0004*) for women. However, because we evaluated model performance in the same data set used to build the risk model, these NRIs may be somewhat overestimated. Moreover, it is unclear whether twice the average lifetime risk is a clinically actionable threshold: only 8.4% of male cases (3.5% of female) have more than twice the average lifetime risk. Most of those identified as high risk will not go on to develop pancreatic cancer, because the average lifetime risks in both men and women are low. Twice the average lifetime risk is 2×1.47% = 2.94% in men and 2×1.31% = 2.62% in women, and 96.3% of men and 96.6% of women above these risk thresholds will *not* develop pancreatic cancer in their lifetimes.

The risk models with and without the genetic variables do not identify subsets of individuals at very high lifetime risks. Using controls to estimate the distribution of risks in U.S. non-Hispanic whites, 4/1,000 men and 2/1,000 women would be classified as having lifetime risk greater than 5%, and none would be classified as having more than 7% lifetime risk.

## Discussion

In this study, we generated a pancreatic cancer risk model based on established non-genetic and genetic risk factors and calculated absolute risks based on relative risk estimates and US incidence. The risk factors considered were smoking, heavy alcohol intake, high BMI, diabetes, family history of pancreatic cancer, ABO non-O blood group and three common genetic variants identified by GWAS. We found that even if all these known risk factors are included in the model, most individuals will only be at modestly increased risks because relatively few individuals have a high number of risk factors. In addition, we found that the genetic factors did not add substantively to a risk model based on life-style factors only, as most individuals remained in the same risk strata.

The low absolute risks observed here for most individuals, together with the current lack of non-invasive and low cost screening tools, argue against screening programs for the general population and underscore the importance of research to identify novel risk markers. Given the very high mortality rate of pancreatic cancer, it remains an open question whether future screening tools could be implemented for individuals in the population who are at the highest risks, for example individuals with estimated lifetime risks above 5%. It is important to note that our model does not account for known high-penetrant genetic variants or strong familial risk. Individuals with a strong family history of cancer may benefit from genetic counseling. For such individuals genetic counseling in conjunction with the PancPRO [38] model can provide individual level risk estimates.

This study is based on data from a series of cohort and case-control studies and constitutes the largest risk model analysis of pancreatic cancer to date. It is also the first risk model for pancreatic cancer that includes non-genetic risk factors. Our model can easily be modified to include any new discovered risk factors.

Our study has several limitations. As with all risk scores that include genetic variants identified from GWAS, we are most likely including proxies for the causative genetic variants. Identification of the causal alleles might result in better performance in our model. Moreover, by focusing on genome-wide significant markers, we are not including markers that are truly associated with pancreatic cancer risk but did not achieve statistical significance. More sophisticated multivariable modeling techniques might be able to use these latent risk markers to improve predictive ability, but these methods greatly increase the risk of overfitting and require sample sizes an order of magnitude larger than the number of cases and controls used in this study [39], [40].

We only measured modifiable risk factors during one point in time. As these risk factors may change over time, our assessment does not completely capture the cumulative lifetime exposure. We categorized continuous variables in order to balance model parsimony and flexibility; however, this approach may have led to a loss of fine-scale information on exposure distribution. The list of non-genetic risk factors included here is not complete and future studies should consider other risk factors. Here we limited the list of non-genetic factors to well-established non-genetic risk factors that were assessed in our study population. For example, information on chronic pancreatitis was not included in these analyses due to limited availability of pancreatitis data from the cohorts and the low prevalence of this disease. We included data from both prospective cohort studies and retrospective case-control studies. For the prospective data exposure information may have changed between data collection and occurrence of pancreatic cancer, while retrospective data can be subject to recall bias. However, the risk estimates were consistent across study designs for all exposures other than diabetes mellitus (Table S3).

Our model does not directly measure absolute risk but rather relies on incidence estimates from the SEER data. We used our controls data to estimate the distribution of risk factors among U.S. non-Hispanic whites. The distribution of risk factors in these controls is likely different than that of the general U.S. population, as cohort participants are likely healthier and risk factors such as smoking are less prevalent, and not all studies were based in the United States. These differences may have affected our risk estimates in several ways. On the one hand, we may have underestimated the proportion of U.S. non-Hispanic whites who would be classified as high risk. On the other hand, by underestimating the average relative risk (which is inversely related to the baseline risk), we may have overestimated risk for individuals with particular genetic and non-genetic profiles. Given that lifetime risk estimates remained quite low (most less than 5% and all less than 7.5%) with little variation across the study population, this possible overestimation does not impact our conclusions on the utility of this model.

Our analysis is based solely on a population of European ancestry, so it cannot be generalized to other ethnicities, some of which have a greater risk of pancreatic cancer [42].

Model fit and reclassification were assessed in the same populations used to obtain the risk estimates for the model; therefore, it is possible that the results presented here overestimate how the risk model would perform in an independent study population. However, we deliberately chose a parsimonious approach to modeling, focusing on well-established risk factors, in order to minimize the risk of overfitting [41]. The risk estimates for non-genetic covariates observed in this study are consistent with the existing literature; thus, we would expect our non-genetic model to perform similarly in other non-Hispanic white populations. Because the genetic risk markers were discovered in this set of samples [27], [28], the per-allele odds ratios for these markers may be overestimated due to the “winner's curse” phenomenon [42]. We used the weighted maximum likelihood method of Zhong and Prentice to adjust for inflation due to winner's curse [43]. The effects at the *ABO* and 13q22 loci were not appreciably inflated; the estimates for rs3790844 at chr1q32.1 and rs401681 at 5p15.33 were slightly inflated, with inflation factors of 2% and 7%, respectively. The AUROCs using the winner's-curse-adjusted per-allele odds ratio estimates change only slightly: AUROC = 0.55 (0.53,0.47) for the model using risk alleles alone (as compared to 0.57) and c = 0.60 (0.58,0.62) for the model with both the risk alleles and clinical risk factors (as compared to 0.61).

In summary, in a large study sample, we derived an absolute- risk model for pancreatic cancer and used our model to estimate risks in the Non-Hispanic White US population. We found that although all risk factors were individually associated with pancreatic cancer, the low frequencies of many of the exposures, along with the small magnitudes of their risks and even that of their aggregated sum resulted in relatively low ten-year absolute risks. Thus, absolute risk modeling can identify a subset of the general population at higher than average risk of pancreatic cancer, but with the risk factors so far considered, the clinical utility of such general population models at this time may be limited.

## Supporting Information

Table S1 **Case-control studies in PanScan.**
(DOC)

Table S2 **Nested case-control studies from cohorts in PanScan.**
(DOC)

Table S3 **Association between pancreatic cancer risk and smoking, personal history of diabetes, family history of pancreatic cancer, alcohol use, body mass index, and known genetic markers stratified by study design (case-control vs cohort).**
(DOCX)
