# Supplementary material for: An Absolute Risk Model to Identify Individuals at Elevated Risk for Pancreatic Cancer in the General Population
Source: PLoS One. 2013 Sep 13;8(9):e72311. doi: 10.1371/journal.pone.0072311 (PMC3772857; doi:10.1371/journal.pone.0072311)
Supplement: Table S1 — Case-control studies in PanScan. (DOC) [file pone.0072311.s001.doc]

| **Study** | **Cases** | **Controls** | **Sources of Cases** | **Sources of Controls** | **Date of case (top) and control (bottom) recruitment** | **Mean age at Diagnosis Cases** | **Mean Age Controls** | **Male %** | **European Ancestry %** | **Matching Criteria** | **Reference** |
| --- | --- | --- | --- | --- | --- | --- | --- | --- | --- | --- | --- |
| University of California San Francisco (UCSF) | 214 | 221 | Population-based cancer registry | Random-digit dialing within 6 SF Bay Area counties, | 1993-1999  1995-1999 | 64.6(10.6) | 64.2 (11.7) | 54.5 | 84.8 | Frequency matched by age and sex | (1) |
| Yale | 185 | 280 | Population-based cancer registry | Block list directed Random digit dialing, no personal history of cancer (excluding non-melanoma skin) | 2004-2008  1999-2003 | 66.9(10.1) | 66.6 (10.2) | 53.8 | 94.2 | Frequency matched by age and sex | (2) |
| Toronto | 279 | 259 | Population-based cancer registry | Ontario population-based case control study of colorectal cancer (ARCTIC) | 2000-2008  1999-2003 | 63.9(10.0) | 62.7(8.9) | 54.8 | 94.6 | Age, sex, ethnicity, no personal history of colorectal cancer | (3) |
| MD Anderson | 228 | 265 | Hospital | Friends and spouses of non-pancreatic cancer patients at MDA, no personal history of cancer (excluding non-melanoma skin) | 1997-2007  2004-2007 | 61.7(9.7) | 60.9 (9.7) | 59.4 | 95.1 | Frequency matched by age, Ethnicity and sex | (4) |
| Johns Hopkins | 185 | 190 | Hospital | Spouse (in-law) of patients, | 1996-2007  1996-2007 | 64.6(10.3) | 68.9 (11.1) | 44.5 | 95.9 | None | (5) |
| Mayo Clinic Molecular Epidemiology Case-Control Study | 510 | 597 | Clinic | Primary Care patients, no personal history of cancer (excluding non-melanoma skin) | 1991-2008  2004-2007 | 65.8(10.5) | 66.3 (10.0)  66.3 (10.1) | 54.7 | 98.6 | Frequency matched by age, Ethnicity, sex and residence | (6) |
| Memorial Sloan Kettering Cancer Center | 129 | 137 | Clinic | Spouses of patients, visitors accompanying patients, no personal history of cancer (excluding non-melanoma skin) | 2002-2008  2004-2008 | 59.5(10.3) | 58.5 (10.6) | 50.0 | 96.4 | None | (7) |
| PACIFIC Study | 276 | 270 | Group Health (Seattle Pufet Sound) and Kaiser Permanente Northern CA | Group Health (Seattle Pufet Sound) and Kaiser Permanente Northern CA | 2005-2008  2005-2008 | 68.7(11.1) | 69.7 (11.3)  69.6 (11.3) | 50.0 | 100 | Frequency matched by Ethnicity, age, enrollment duration in HMO and sex | (8) |

**Table S1:** Case-control studies in PanScan

**REFERENCES**

1. Duell,E.J. *et al.* Detecting pathway-based gene-gene and gene-environment interactions in pancreatic cancer. *Cancer Epidemiol. Biomarkers Prev.* **17**,

1470-1479 (2008).

2. Risch,H.A. Etiology of pancreatic cancer, with a hypothesis concerning the role of N-nitroso compounds and excess gastric acidity. *J. Natl. Cancer Inst.* **95**,

948-960 (2003).

3. Eppel,A., Cotterchio,M., & Gallinger,S. Allergies are associated with reduced pancreas cancer risk: A population-based case-control study in Ontario,

Canada. *Int. J. Cancer* **121**, 2241-2245 (2007).

4. Hassan,M.M. *et al.* Risk factors for pancreatic cancer: case-control study. *Am. J. Gastroenterol.* **102**, 2696-2707 (2007).

6. McWilliams,R.R. *et al.* Polymorphisms in DNA repair genes, smoking, and pancreatic adenocarcinoma risk. *Cancer Res.* **68**, 4928-4935 (2008).

7. Olson,S.H. *et al.* Allergies, variants in IL-4 and IL-4R alpha genes, and risk of pancreatic cancer. *Cancer Detect. Prev.* **31**, 345-351 (2007).
