# Supplementary material for: An Absolute Risk Model to Identify Individuals at Elevated Risk for Pancreatic Cancer in the General Population
Source: PLoS One. 2013 Sep 13;8(9):e72311. doi: 10.1371/journal.pone.0072311 (PMC3772857; doi:10.1371/journal.pone.0072311)
Supplement: Table S2 — Nested case-control studies from cohorts in PanScan. (DOC) [file pone.0072311.s002.doc]

| **Study** | **Cases** | **Controls** | **Enrollment** | **Mean age at Diagnosis Cases** | **Mean Age Controls** | **Male %** | **European Ancestry (%)** | **Matching criteria** | **Reference** |
| --- | --- | --- | --- | --- | --- | --- | --- | --- | --- |
| The Alpha-Tocopherol Beta-Carotene Prevention Study (ATBC) | 194 | 206 | 1985-1988 | 70.5 (5.6) | 70.5(5.6) | 100 | 100 | Ethnicity, sex, age, date of blood draw, alive at time case diagnosed | (1) |
| Give us a Clue to Cancer and Heart Disease Study (CLUEII) | 63 | 67 | 1989 | 69.3(11.5) | 69.2 (10.9) | 47.7 | 100 | Ethnicity, sex, age, date of blood draw, alive at time case diagnosed | (2) |
| Cancer Prevention Study (CPSII) | 118 | 115 | 1992-1993 | 75.1(5.8) | 75.1 (5.8) | 56.7 | 97.9 | Ethnicity, sex, birth year, DNA source(blood or buccal), alive at time case diagnosed | (3) |
| European Prospective Investigation Into Cancer and Nutrition Study (EPIC) | 418 | 436 | 1988-2002  87-02 | 62.1(7.8) | 65.9 (7.8) | 46.5 | 100 | Sex, center, date of blood draw, alive at time case diagnosed | (4) |
| Health Professionals Follow-Up Study(HPFS) | 53 | 51 | 1993-1995  1986 | 71.1(7.8) | 71.8 (7.6) | 100 | 100 | Sex, age, date of blood draw, alive at time case diagnosed, smoking status (never/former/current) | (5) |
| Nurses’ Health Study | 72 | 80 | 1989-1990  1976 | 69.9(6.4) | 68.6 (7.04)  68.6 (7.1) | 0 | 91.6 | Sex, age, date of blood draw, alive at time case diagnosed, smoking status (never/former/current) | (6) |
| The New York University Women’s Health Study (NYU-WHS) | 9 | 10 | 1988-1990  85-87 | 69.4(9.8) | 69.4 (8.8) | 0 | 76.9 | Ethnicity, sex, age, date of blood draw, alive at the time the case was diagnosed, menopausal status at enrollment | (7) |
| Physicians Health Study (PHS) | 29 | 52 | 1982-1983 | 75.3(7.5) | 67.8 (9.1) | 100 | 79.6 | Ethnicity, sex, age, date of blood draw, alive at time case diagnosed, smoking status (never/former/current) | (8) |
| Prostate, Lung, Colorectal Ovarian Cancer Screening Trial (PLCO) | 182 | 202 | 1994-2001 | 71.6(5.9) | 71.5 (6.2) | 60.4 | 91.7 | Ethnicity, sex, age, birth year, date of blood draw, alive at time case diagnosed, DNA source (blood or buccal), study arm and center | (9) |
| Shanghai Men’s and Women’s Health Study (SMWHS) | 61 | 65 | 1996(Women)  2002 (Men) | 66.3(7.4) | 66.6 (6.9) | 20.6 | 0 | Sex, age, birth year, menopausal status at baseline, date of blood draw, alive at the time the case was diagnosed | (10,11) |
| Women’s Health Initiative (WHI) | 184 | 196 | 1992-1998 | 72.3(7.4) | 71.8 (7.5) | 0 | 85.5 | Ethnicity, sex, age, center, enrollment date, study arm, hysterectomy status, menopausal status, alive at the time the case was diagnosed | (12) |
| Women’s Health Study (WHS); | 21 | 30 | 1992-1993 | 60.6(7.4) | 62.5(7.20) | 0 | 94.7 | Ethnicity, age, birth year, smoking status (never, former/current) date of blood draw, alive at the time the case was diagnosed | (13) |

**Table S2:** Nested case-control studies from cohorts in PanScan

**REFERENCES**

1. The alpha-tocopherol, beta-carotene lung cancer prevention study: design, methods, participant characteristics, and compliance. The ATBC Cancer

Prevention Study Group. Ann Epidemiol 1994;4:1-10.

2. Gallicchio L, Chang H, Christo DK, et al. Single nucleotide polymorphisms in inflammation-related genes and mortality in a community-based cohort in Washington County, Maryland. Am J Epidemiol 2008;167:807-13.

3. Calle EE, Rodriguez C, Jacobs EJ, et al. The American Cancer Society Cancer Prevention Study II Nutrition Cohort: rationale, study design, and baseline characteristics. Cancer 2002;94:2490-2501.

4. Riboli E, Hunt KJ, Slimani N, et al. European Prospective Investigation into Cancer and Nutrition (EPIC): study populations and data collection. Public Health Nutr 2002;5:1113-24.

5. Wei EK, Giovannucci E, Fuchs CS, Willett WC, Mantzoros CS. Low plasma adiponectin levels and risk of colorectal cancer in men: a prospective study. J Natl Cancer Inst 2005;97:1688-94.

6. Colditz GA, Hankinson SE The Nurses' Health Study: lifestyle and health among women. Nat Rev Cancer 2005;5:388-96.

7. Zeleniuch-Jacquotte A, Gu Y, Shore RE, et al. Postmenopausal levels of sex hormones and risk of breast carcinoma in situ: results of a prospective study. Int J Cancer 2005;114:323-7.

8. Ma J, Giovannucci E, Pollak M, et al. A prospective study of plasma C-peptide and colorectal cancer risk in men. J Natl Cancer Inst 2004;96:546-53.

9. Hayes RB, Sigurdson A, Moore L, et al. Methods for etiologic and early marker investigations in the PLCO trial. Mutat Res 2005;592:147-54.

10. Zheng W, Chow WH, Yang G, et al. The Shanghai Women's Health Study: rationale, study design, and baseline characteristics. Am J Epidemiol

2005;162:1123-31.

11. Xu WH, Zhang XL, Gao YT, et al. Joint effect of cigarette smoking and alcohol consumption on mortality. Prev Med 2007;45:313-19.

12. Anderson GL, Manson J, Wallace R, et al. Implementation of the Women's Health Initiative study design. Ann Epidemiol 2003;13:S5-17.

13. Rexrode KM, Lee IM, Cook NR, Hennekens CH, Buring JE. Baseline characteristics of participants in the Women's Health Study. J Womens Health Gend Based Med 2000;9:19-27.
