# Supplementary material for: An Absolute Risk Model to Identify Individuals at Elevated Risk for Pancreatic Cancer in the General Population
Source: PLoS One. 2013 Sep 13;8(9):e72311. doi: 10.1371/journal.pone.0072311 (PMC3772857; doi:10.1371/journal.pone.0072311)
Supplement: Table S3 — Association between pancreatic cancer risk and smoking, personal history of diabetes, family history of pancreatic cancer, alcohol use, body mass index, and known genetic markers stratified by study design (case-control vs cohort). (DOCX) [file pone.0072311.s003.docx]

**Table S3.** Association between pancreatic cancer risk and smoking, personal history of diabetes, family history of pancreatic cancer, alcohol use, body mass index, and known genetic markers stratified by study design (case-control vs cohort)

|  | **Multivariate Odds Ratio (95% CI)** | |
| --- | --- | --- |
| **Characteristic** | **Case-Control*** | **Cohort*** |
|  |  |  |
| **Cigarette smoking**  Never  Former  Current | 1.00  1.29 (1.12,1.50)  2.46 (1.96, 3.08) | 1.00  1.07 (0.88,1.30)  1.83 (1.38, 2.43) |
|  |  |  |
| **Diabetes mellitus**  Never  >3 years duration  Unknown | 1.00  1.77 (1.37,2.28)  1.10 (0.75, 1.36) | 1.00  1.61(1.16,2.26)  1.91 (1.37, 2.26) |
| **Family history of pancreatic cancer**  No  Yes | 1.00  1.55(1.16,2.15) | 1.00  1.83 (1.03, 3.25) |
| **Heavy alcohol use (> 3 drinks per day)**  No  Yes | 1.00  1.56(1.22, 2.00) | 1.00  1.31 (0.96, 1.80) |
| **Body mass index**  <18.5  18.5-25  25-30  >30 | 1.13(0.57, 2.27)  1.00  1.01(0.86, 1.19)  1.39(1.14, 1.70) | 0.72 (0.32, 1.60)  1.00  1.18(1.00, 1.40)  1.11(0.90, 1.37) |
| **ABO genotype**  OO  AO  AA  BO  BB  AB | 1.00  1.18(1.03, 1.36)  1.32(1.04, 1.68)  1.30 (1.05, 1.60)  1.10(0.57, 2.14)  1.50 (1.10, 2.05) | 1.00  1.32(1.11, 1.58)  1.79(1.34, 2.38)  1.46(1.13, 1.88)  2.52(1.18, 5.39)  1.47(1.05, 2.05) |
| **1q32 rs3790844 (per risk allele)** | 1.22(1.08-1.34) | 1.34(1.21,1.49) |
| **5p15 rs401681 (per risk allele)** | 1.06( 0.96-1.18) | 1.26(1.15,1.37) |
| **13q22 rs9543325 (per risk allele)** | 1.24 (1.11–1.39) | 1.28 (1.17, 1.40) |
